# One Health Surveillance for Rabies: A Case Study of Integrated Bite Case Management in Albay Province, Philippines

**DOI:** 10.3389/fitd.2022.787524

**Published:** 2022-03-31

**Authors:** Kristyna Rysava, Jason Espineda, Eva Angela V. Silo, Sarah Carino, Ariane Mae Aringo, Rona P. Bernales, Florencio F. Adonay, Michael J. Tildesley, Katie Hampson

**Affiliations:** 1 The Zeeman Institute for Systems Biology and Infectious Disease Epidemiology Research, School of Life Sciences and Mathematical Institute, University of Warwick, Coventry, United Kingdom; 2 Department of Agriculture Regional Field Office 5, Regional Animal Disease, Diagnostic Laboratory, Camalig, Philippines; 3 Albay Veterinary Office, Camalig, Philippines; 4 Institute of Biodiversity, Animal Health and Comparative Medicine, University of Glasgow, Glasgow, United Kingdom

**Keywords:** rabies surveillance, one health, IBCM, intersectoral collaboration, rabies management

## Abstract

Canine rabies is a significant public health concern and economic burden in the Philippines. Animal Bite Treatment Centers (ABTCs) that provide post-exposure prophylaxis (PEP) to bite patients have been established across the country, but the incidence of bite patient presentations has grown unsustainably, whilst rabies transmission in domestic dogs has not been controlled. Moreover, weak surveillance leads to low case detection and late outbreak responses. Here we investigated the potential for Integrated Bite Case Management (IBCM) to improve rabies detection in Albay province. Using information obtained from animal bite histories combined with phone follow-ups and field investigations, we demonstrated that IBCM resulted in a fourfold increase in case detection over 13 months of study compared to the prior period. Bite patient incidence across Albay was very high (>600/100,000 persons/year) with PEP administered mostly indiscriminately. Clinic attendance reflected availability of PEP and proximity to ABTCs rather than rabies incidence (<3% of patient presentations were from “probable” or confirmed rabies exposures) and is therefore not a suitable indicator of rabies burden. Further analysis of the IBCM data suggests that rabies transmission is mostly localized with focal cases from the previous month and current cases in neighbouring villages being most predictive of future rabies occurrence. We conclude that investigations of suspicious biting incidents identified through IBCM have potential to foster intersectoral relationships, and collaborative investments between public health and veterinary services, enabling the One Health ethos to be applied in a more sustainable and equitable way. Triage of patients and investigations of suspect dogs offer an effective tool for improved PEP provisioning and reduction of unnecessary expenditure, whilst targeted field investigations should lead to increased and earlier detection of rabid dogs. Given the enduring risk of re-introductions from neighbouring populations, enhanced surveillance is critical to achieving and maintaining rabies freedom.

## Introduction

Canine rabies has been eliminated across the Global North, but throughout the majority of low- and middle-income countries (LMICs), the disease remains a serious public health concern and economic burden. Every year, thousands of people die, and billions of dollars are lost due to rabies spread by domestic dogs (1). Although fatal once symptoms appear, rabies is preventable through prompt administration of post-exposure prophylaxis (PEP) after a person is bitten by a rabid animal, and the virus can be eliminated from source populations through mass dog vaccination (2). PEP is highly effective in preventing human deaths, but shortages, high costs and limited awareness of rabies in communities still leaves many people at risk. Elimination of rabies through mass dog vaccination can negate these risks and is ultimately a more effective solution. However, to date there are only a few LMICs where dog vaccination has been sufficiently scaled up and sustained to achieve this goal (3).

In the Philippines, rabies control and prevention efforts are being implemented, but only a few islands and provinces are on track for elimination of both human and dog rabies (4, 5). Despite ongoing initiatives by the national government to interrupt transmission and prevent the disease, canine rabies remains a major public health challenge, with 200-300 people dying from rabies every year (6). Though there is generally high rabies awareness in the Philippines, elevated through anti-rabies initiatives, human rabies deaths are still occurring and consequently the demand for PEP is continuing to increase. As a result, local and national healthcare budgets are under strain from the expensive administration of often unnecessary PEP, which in turn results in frequent vaccine shortages and redirects focus away from mass dog vaccination campaigns.

The lack of effective and integrated cross-sectoral surveillance is a major impediment to understanding local rabies dynamics and effectively controlling the disease, with relatively sparse detection of rabies cases in dogs (even where human cases are diagnosed). Estimates suggest that less than 10% of canine rabies cases are detected in many endemic settings where routine surveillance is rudimentary (7, 8). Current routine surveillance guidelines in the Philippines recommend that an investigation is only carried out when a human rabies case is detected (9; personal communications with Dr Florencio Adonay and Dr Rona Bernales). However, by this time it is too late to detect the rabid dog responsible for the case, perhaps explaining why so few dog cases are diagnosed in the country. Conversely, active case-finding by tracing dog-bite patients attending Animal Bite Treatment Centers (ABTCs) can improve surveillance (6). Histories of biting animals provide valuable and oftentimes the only epidemiological information to inform rabies management. Where animal rabies surveillance is very weak, information on the history of the biting animal from bite-patient presentations may provide a better measure of disease incidence than laboratory confirmed cases and can be used to guide investigations of rabies suspect incidents. Moreover, contact-tracing of transmission events linked to bite-patients presenting at ABTCs allows reconstruction of rabies transmission pathways that are in turn critical to identification of areas where dog vaccination requires improvement. Ultimately, integrated approaches for rabies surveillance using information collected by interviewing dog-bite patients to initiate investigations of biting animals – Integrated Bite Case Management (IBCM) – has potential to increase case detection and expedite outbreak response, while strengthening intersectoral relationships between public health and veterinary services, including trust in data sharing and prospects for integrated surveillance systems (10).

Here we present a descriptive epidemiological study of an integrated intersectoral surveillance approach aiming to link human and animal health sectors to guide investigations in response to suspicious animal bite incidents. Specifically, we developed detailed epidemiological questionnaires, recorded using a mobile phone application at three ABTCs in Albay province, Philippines to document bite incidents, and to inform and trigger field investigations for timely detection and containment of rabies in dogs. Our objectives were 1) to examine the relationship between bite patient presentations and rabies cases; 2) to determine whether the histories of bite incidents can be a reliable predictor of rabies risk and therefore 3) to compare our approach to the existing routine surveillance and assess whether IBCM can improve detection of rabies in the dog population.

## Materials and Methods

### Study Site

We established a 13-month longitudinal study of dog bite-injury patients beginning in March 2018 in Albay province, in central Bicol peninsula (Region V, Philippines; Figure 3A). The province comprises 19 municipalities and a human and dog population of 1,314,826 (11) and 161,478 (Albay Provincial Veterinary Services) in 2015 respectively. Rabies in the province likely persists at a low-level of endemicity with frequent incursions from adjacent provinces and nearby islands, reflecting dynamics from across the Philippines archipelago (4, 12). Dog vaccination has been conducted intermittently in the region since 2013. Between 2015 and 2016 annual dog vaccination campaigns were strengthened through the OIE STANDZ (World Organisation for Animal Health Stop Transboundary Animal Diseases and Zoonoses) project (5, 13), but have since become less comprehensive and more spatially heterogeneous, with some barangays (villages) receiving no vaccines in certain years. The average cost per dog vaccination in the Philippines using parenteral vaccination is ~2 USD (4). Data on mass dog vaccination were provided by Albay Provincial Veterinary Services (PVS) office and data on the human population were taken from the Philippine Statistics Authority official website (11).

### PEP Provision

Both rabies post-exposure vaccines and immunoglobulins (RIG) are provided to bite patients free-of-charge from government-run ABTCs located within hospitals and can also be bought privately (at a cost of 600-1000 PHP/12-20 USD and 1300-2000 PHP/25-40 USD per dose of Rabipur and Verorab respectively) from Animal Bite Clinics (ABCs). ABTCs administer vaccines intradermally (ID) following the updated Thai Red Cross regimen (TRC) with two 0.1ml doses (to deltoids) delivered on day 0, 3, 7 and 28 (14); however, patients are typically charged for the fourth dose (~650 PHP/13 USD per dose). Following the WHO guidelines (15), RIG, if available, should be provided to all Category III bite patients, directly into and around the wound and as soon as possible after the exposure; however, delays are common as clinics usually store only small amounts of RIG if any at all. The exact dose of RIG depends on the weight of the patient, with approximately 2 ml (equal to one vial) required per 10 kg (16) resulting in a typical cost per patient of around 5400 PHP/106.5 USD for RIG. There are three government-run ABTCs in Albay province located at Bicol Regional Training and Teaching Hospital (BRTTH) in Legazpi City, Josefina Belmonte Duran Memorial District Hospital (JBDMDH) in Ligao City and Ziga Memorial District Hospital (ZMDH) in Tabaco City (Figure 3A). The only private ABC in Albay is located in Daraga, a short distance from Legazpi City, typically visited less frequently by patients than ABTCs due to the additional costs of purchasing PEP.

### Bite Patient Interviews and Follow-Up

From 1 March 2018 to 31 March 2019, one data collector was stationed at each of the three ABTCs. The ABC in Daraga was excluded due to negligible patient throughput. At each ABTC nurses recorded bite patient information including their name, address, and phone number within standardized animal bite registers, which were shared with the data collectors who conducted a short interview with patients after PEP was administered. During the interviews the data collectors recorded details of the bite incident, including the history and health condition of the biting animal and of the exposure and treatment (Interview Forms Figure S1).

All patients were requested to ensure that the biting dog would be put in quarantine and observed for 14 days, either by managing their own dogs directly or by contacting the dog owner to follow the same protocol. Additionally, bite patients/dog owners responsible for their dogs were urged to immediately contact the data collector should any behavioural/symptomatic changes occur during the quarantine period. Otherwise, when telephone numbers were provided, bite victims were called after 14 days, and a follow-up questionnaire was completed over the phone to identify whether the biting animal was rabid or healthy. Criteria used for this assessment were as follows: if the animal was either sick, exhibited unusual aggression, had bitten multiple people/animals, had been killed, died or was untraceable during the 14 days following the bite. If any or any combination of these criteria were reported during the 14-day period a field investigation was prompted by contacting a corresponding Local Governmental Unit (LGU) officer (Investigation Guidelines Figure S2). Specifically, a field investigation performed by an LGU officer was immediately prompted for: unvaccinated dogs that were either exposed to, or a puppy of, a rabid dog, dogs that were symptomatic for rabies, and dogs that died or were killed within the 14 days window. If the suspect animal was found sick or dead, the animal head/brain sample was collected by a trained veterinarian and sent to the Regional Animal Disease Diagnostic Laboratory (RADDL) of the Department of Agriculture Bicol in Cabangan, Camalig (municipality in Albay) for laboratory diagnosis by FAT (Fluorescent Antibody Test).

Investigated incidents were classified either as “Healthy”, “Non-traceable”, “Rabies suspect”, “Rabies probable” or “Rabies confirmed” according to the protocol (Figures 2B, S1). Specifically, dogs that tested rabies positive by FAT were classified as “Rabies confirmed”. Where sample collection was not possible, we classified the investigated dogs as “rabies probable”. Dogs that disappeared before a field investigation could be initiated but showed aggressive behaviour/unprovoked biting were considered “rabies suspect”. Unvaccinated dogs that did not show any clear symptoms at the time of the biting incident and could not be traced after the 14-day observation period were classified as “non-traceable”. All remaining dogs, both vaccinated and non-vaccinated, that showed no behavioural/symptomatic changes after the 14-day quarantine period were considered “Healthy”. Government-led routine field investigations were also conducted if a human rabies death occurred (9).

**Figure 1:**
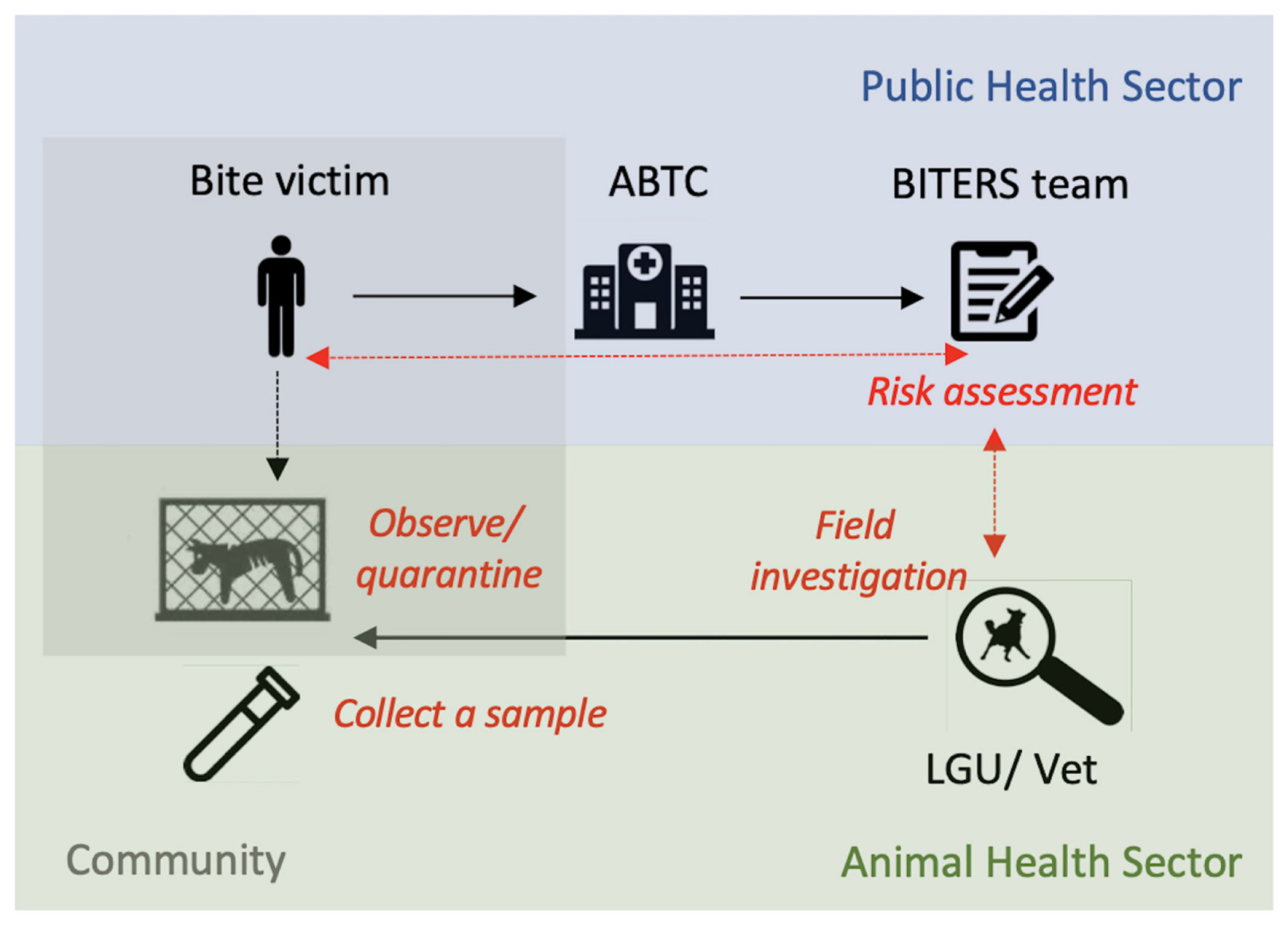
Example of a workflow for intersectoral surveillance approach. Blue shading indicates activities managed by the public health sector, green represents activities falling under the animal health sector and grey shading represents measures that could be undertaken by the communities. Black solid arrows show the workflow, whereas red dashed arrows represent communication between sectors. Bite victims seek care at ABTCs. Upon PEP administration, patient’s details are recorded, followed by an interview with a data collector who conducts a risk assessment based on the history of the bite and biting animal. If considered to be high risk, a designated LGU/veterinary officer receives an alert to initiate a field investigation. All patients are advised to quarantine/observe the biting animal, and after a 14-day quarantine period are contacted over the phone by the data collector to provide follow-up information on potential changes in the animal’s health condition/behaviour. If relevant, this information is then communicated to the LGU/veterinary personnel to initiate a field investigation to confirm the animal status and where available, collect a brain sample for laboratory testing.

Clinic registry data, patient interviews and phone follow-up information as well as, field investigations, case classification and laboratory results were recorded using a tailor-made mobile phone-based application (BITERS – Bite Incidence Tool for Enhanced Rabies Surveillance; Figure S3). All bite patient cases were initially considered for phone follow-up after 14 days of observation; however, in the instance of the bite incident being entirely provoked by the victim and the biting animal having been vaccinated in the last 12 months (or exclusively kept indoors), the patient/animal owner was advised to observe the animal’s behaviour and report back should any changes occur as opposed to being actively followed up on in 14 days. For all other cases, a strict quarantine was recommended, and for individuals classified as “rabies probable” a relevant LGU officer was contacted to initiate a prompt field investigation. The workflow of the data collection is summarized in Figure 1.

Ethical approval was granted locally by the Institutional Review Board at the BRTTH, overseen by the Philippine Health Research Ethics Board (PHREB), and in the UK by the Biomedical and Scientific Research Ethics Committee (BSREC). All interviewed participants provided a written informed consent, and personal data collected from bite patients were anonymized upon case submission to the server maintained by the University of Warwick.

### Data Analysis

We used the longitudinal data on patient and dog bite histories to evaluate monthly throughput of patients at the ABTCs, and to categorize the proportion of patients completing PEP.

To investigate whether the reported incidence of bite patient presentations predicts dog rabies cases, we conducted a linear Poisson regression model using the laboratory confirmed rabies cases as a response variable. To explore potential drivers of reported bite incidence at the barangay level, we tested for a correlation with distance to ABTCs (Euclidean distance to the ABTC from the centroid of patients’ home barangay) using a linear regression with Gamma errors and an inverse link function, with PEP shortages using linear regression with Poisson errors and with human population density using linear regression with Gaussian errors.

In order to assess the effectiveness of IBCM for dog rabies detection, we tested for correlation between the number of dogs classified as “probable rabies” and the number of laboratory confirmed rabies cases in each barangay using a generalized linear model with Poisson errors and a log link function. To estimate the odds of finding a laboratory confirmed rabies case (i.e., presence-absence) based on the number of identified “probable rabies” cases, we conducted a logistic regression. To assess the predictive power of the logistic model, we calculated the area under curve (AUC), with values above 0.5 and 0.7 indicating an acceptable and good model fit respectively, although not necessarily the absolute fit. This follows a similar approach to Gorsich et al. (17) and enables us to utilise a well-recognised and easily interpretable assessment metric of the predictive ability of our model.

Lastly, we employed the “probable rabies” case data identified by month and barangay to estimate their predictive power of future rabies occurrence. Specifically, we used logistic regression to model the probability that in a given month a barangay will have at least one laboratory confirmed case based on the number of “probable” cases in the current and previous month (given that the mean generation interval of rabies is ~25 days as in 18), and from across four spatial scales: local barangay (note, this variable was possible only for cases from a previous month in that barangay), local neighbourhood (including neighbouring barangays), municipality and province. Model selection was conducted using backwards elimination based on the Akaike Information Criterion (AIC), with the final model selected when no additional terms could be removed.

We used the population and vaccination data sources to reconstruct spatially refined maps of bite incidence per 1,000 persons/barangay/13 months and rabies presence, and to evaluate the relationship between rabies burden across the province, dog vaccination coverage, and proximity to clinics. To estimate monthly vaccination coverage in each barangay, we projected monthly dog population sizes using the standard logistic growth model, with population growth defined as $r=\frac{\text{log}(\frac{N}{N_{0}})}{T}$ where *N* and *N*_0_ stand for final and initial population sizes, respectively and denotes the months between two time points. We used the growth rate to project dog population sizes, *N*_*t*_, at monthly timesteps between March 2018 and March 2019 using dog count data recorded annually by the Albay PVS during mass dog vaccination campaigns. For barangays with missing dog counts we drew the local dog population size from a distribution of barangay-level population sizes within the home municipality. We estimated 183,561 and 210,698 dogs in 2018 and 2019 respectively.

In several barangays, the number of dog vaccines (Rabisin^®^, Boehringer-Ingelheim) reported as used during the campaigns exceeded the estimated dog population size. This often occurs when there is no visible differentiation between vaccinated and unvaccinated dogs and an individual is likely to receive multiple doses during the same campaign. For barangays that reported >85% vaccination coverage upon campaign completion, we assigned the vaccines proportionally to the overall number of dogs and vaccines reported for the given month and barangay with the probability of getting vaccinated equal to $1-(\frac{\left(N_{t}-1\right)}{N_{t}})D_{t}$ where *D*_*t*_ stands for the number of vaccine doses reported as used in a given month. As such the probability of being revaccinated (i.e., the same dog receives two doses) during the same campaign increases as the number of vaccines used approaches the total dog population.

Vaccinations conducted more than six months after the last campaign were treated as annual dog vaccination campaigns. Vaccination coverage *V*_*t*_ (at the timestep *t*) was assumed to wane exponentially at rate *v*, based on the average duration of the vaccine-induced immunity *w* where $v=\frac{1}{w}$ (*w* value taken from 19) and the demographic turnover of the dog population bound by the birth rate *b* and death rate *d* (parameter values from 20), yielding the following formula for waning coverage: *V*_*t*+Δ*t*_ = *V*_*t*_*e*^−(*b*+*d*+*v*)Δ*t*^. For barangays in which repeat vaccination occurred within six months of the previous campaign, we allocated vaccines to previously unvaccinated dogs such that $V_{t+\Delta t}=V_{t}e^{-(b+d+v)\Delta t}+(1-V_{t}e^{-(b+d+v)\Delta t})\frac{D_{t}}{N_{t}}$.

## Results

### Bite Patient Presentations and Relationship to Rabies Cases

Bite incidence in Albay province was very high (685/100,000 persons/13 months) with PEP administered mostly indiscriminately of the WHO (World Health Organization) bite category. A temporary vaccine shortage from April 2018 through to June 2018 led to 911 patients being referred to the private ABC in Daraga where the vaccine was available but not provided free-of-charge. All patients treated at either of the ABTC facilities received at least 1 vaccine dose (8162 patients in total), 82% of patients received 2 doses and 73% patients received 3 doses. Only 17% of patients received the fourth dose, likely due to the cost patients are charged for the last dose. When tabulated by WHO bite category, second and third dose was administered (in this order) to 61% and 57% of patients classified as Category I, 85% and 77% of Category II patients, and 69% and 61% of Category III patients. Additionally, 23% of patients (1887) received RIG.

Ninety percent of patients presenting at ABTCs reported dog bites as their primary injury, followed by 24% reporting scratches and <1% reporting open wound exposures to dog’s saliva (several patients reported multiple injuries of different types). Three patients received a full course of PEP after exposure to a “probable” human rabies case; however, no skin rupture occurred. Whilst most patients adhered to the WHO recommended bite management guidelines – washing their wounds thoroughly with water and soap for 15 minutes (indicating widespread awareness on rabies prevention) – a large number also used traditional medicine (i.e., tandok, tambal and herbs).

**Figure 2:**
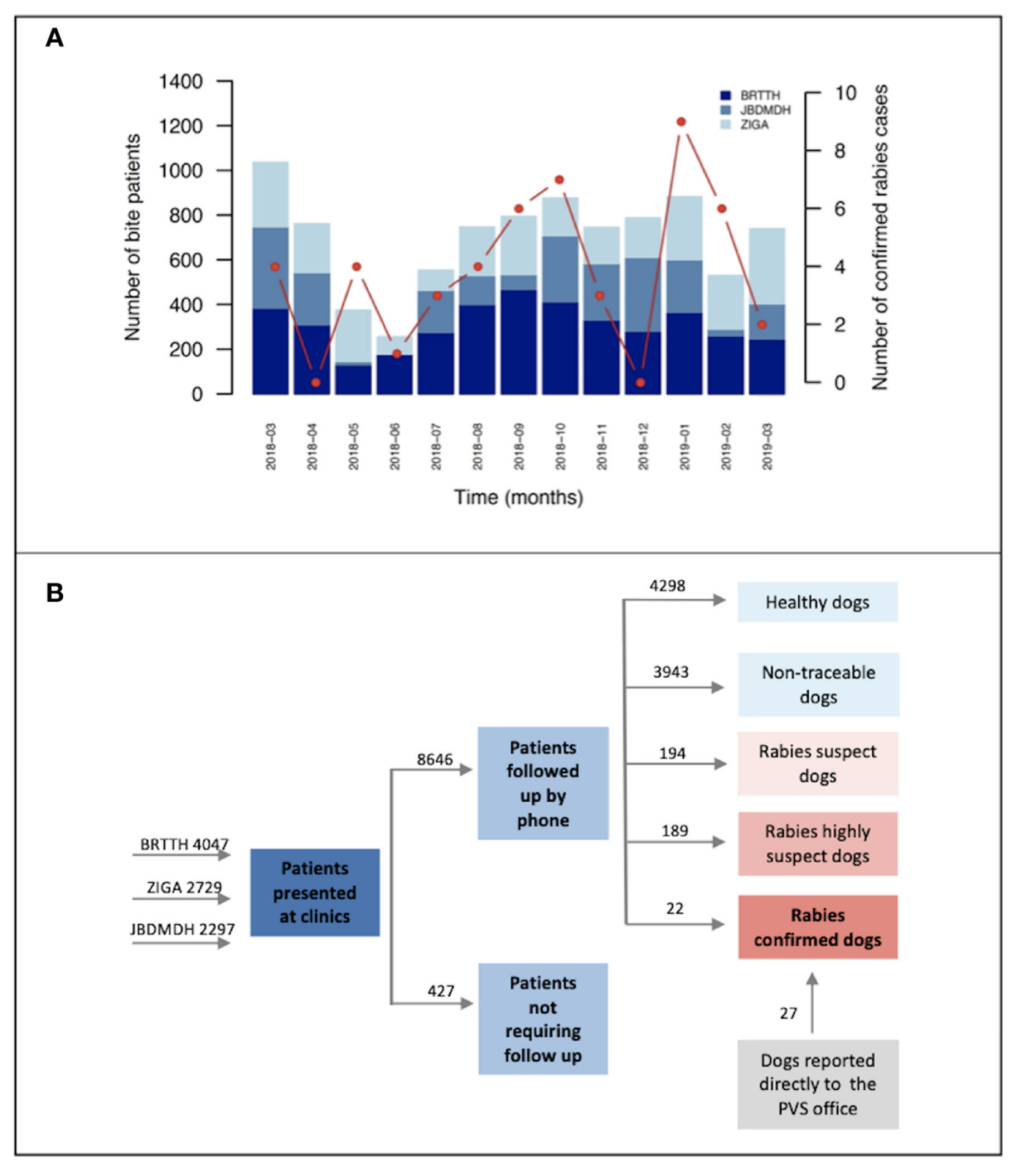
Bite incidence and classification. **(A)** Monthly patient throughput by clinic. Monthly patients presenting at ABTCs in Albay province between March 2018 and March 2019. Red points connected by line segments indicate dog rabies cases confirmed each month with a representative y-axis on the right side of the plot. **(B)** Investigation breakdown from recording of bite victims at the ABTCs to completion of investigations and case classification. Numbers above arrows indicate the sum of cases in each category. BRTTH, Bicol Regional Training and Teaching Hospital in Legaspi City; ZIGA, Ziga Memorial District Hospital in Tabaco City; JBDMDH, Josefina Belmonte Duran Memorial District Hospital in Ligao City.

The total number of patients presenting at the ABTCs varied significantly across months and showed no statistically significant relationship with the number of laboratory-confirmed rabies cases (regression coefficient = 0.0009, p-value = 0.198, residual deviance = 27.25 on 11 degrees of freedom). Instead, the observed pattern likely reflects the vaccine shortage during the first quarter of the year (Poisson regression coefficient = -0.476, p-value < 0.001, residual deviance = 512.42 on 11 degrees of freedom) (Figure 2A).

### Histories of Bite Incidents as a Predictor of Rabies Risk

The vast majority of bites (76%, n=6906) were reported as provoked by the bite victim, with 4298 animals found unequivocally healthy (i.e., no other classification could be applied) after the 14-day observation period. On the basis of the initial patient triage and phone call follow-up we identified 256 incidents (222 dogs as several patients were bitten by the same animal) classified as “rabies probable” of which 6 patients were from the neighbouring province, Sorsogon, and one patient was from Nueva Ecijia, a landlocked province in central Luzon. For 33 of these “rabies probable” dogs we conducted epidemiological field investigations (as described in the Methods); laboratory diagnostics confirmed rabies in 22 of these animals whilst the other 11 tested negative.

**Figure 3:**
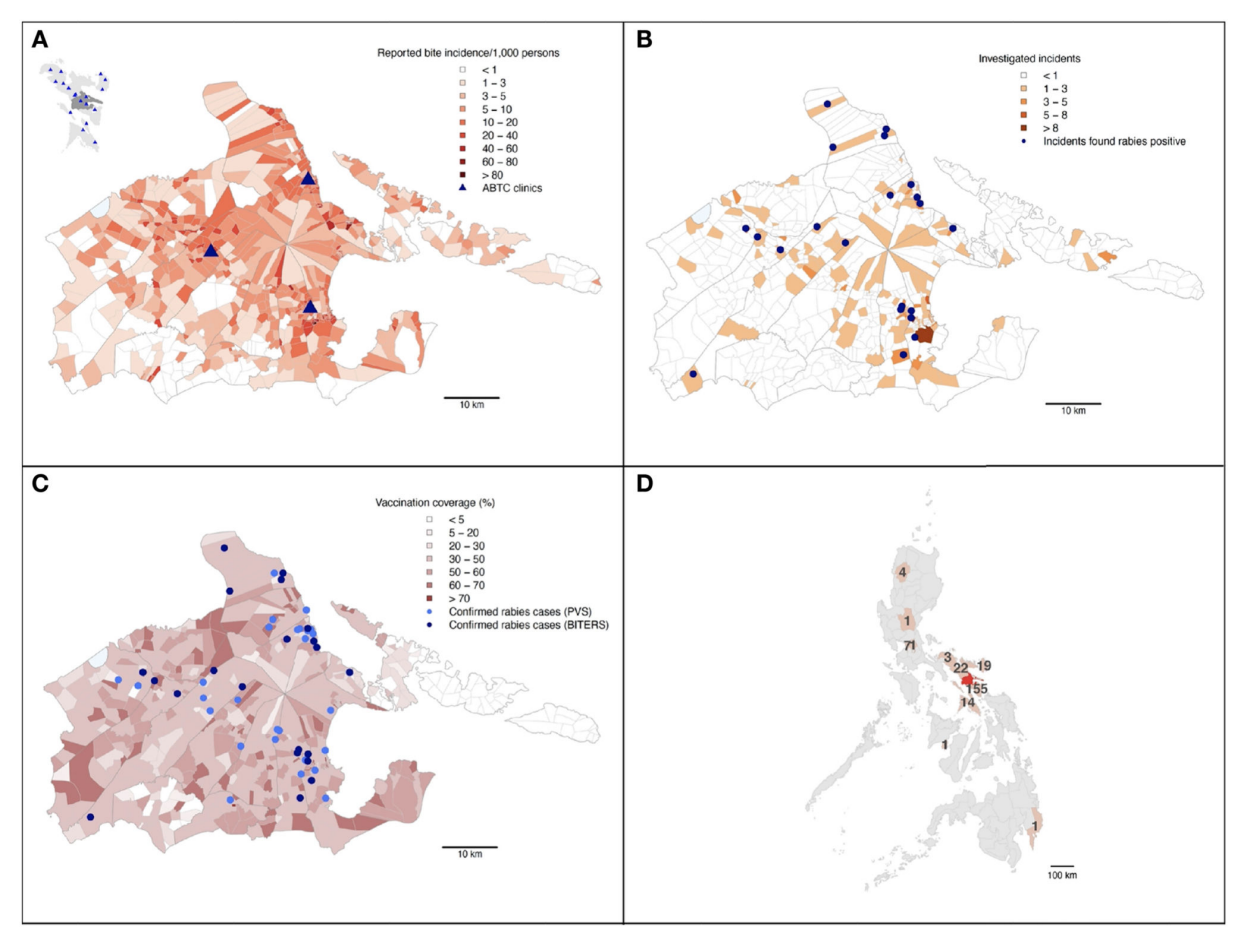
Summary maps of the BITERS surveillance data from Albay province. Albay province shown in **(A)** (with Bicol region as an inset in the top left corner; Albay province indicated by darker grey), **(B, C)**. The Philippine archipelago shown in **(D). (A)** By barangay bite incidence reported at the three ABTCs in Albay province from March 2018 through to March 2019. The inset shows locations of ABTCs (blue triangles) across the Bicol region (lighter grey) with Albay province shaded dark grey. **(B)** Barangays are shaded by the number of bites identified and referred for field investigations through patient triage. Blue points show locations of incidents involving dogs that were found rabies confirmed upon investigation. **(C)** All rabies confirmed dog cases (identified by either the BITERS team or the PVS differentiated as lighter and darker blue points respectively) in relation to average dog vaccination coverage in barangays over the study period. **(D)** The number of bite patients who presented at an ABTC in Albay following being bitten outside of the province. Provinces of the exposures and Albay shown in light and dark red respectively.

**Figure 4:**
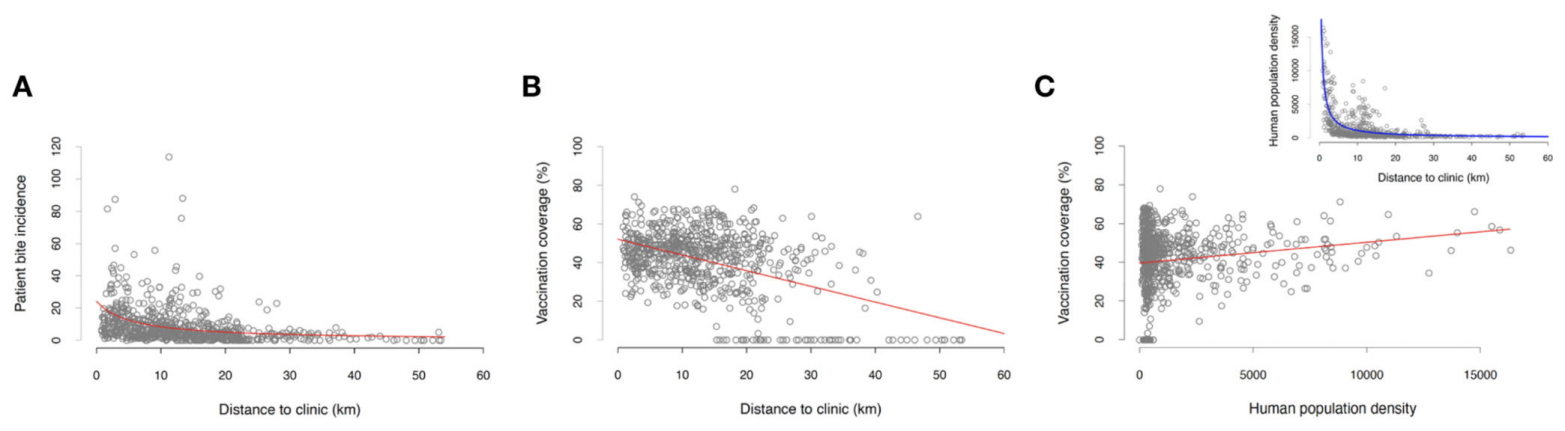
Summary regression analyses using BITERS surveillance data shown as grey points with lines indicating the fitted relationship based on the model output. Relationships between **(A)** barangay-level bite incidence and distance to the ABTC, **(B)** dog vaccination coverage (projected monthly and averaged across the study period) and the distance to the ABTC, and **(C)** dog vaccination coverage (projected monthly and averaged across the time period of the study) and the population density. Inset in **(C)** shows the relationship between barangay population density and the distance to ABTCs; locations of ABTC are located in highly populated urban areas that tend to achieve higher dog vaccination coverage. Statistics of all reported models summarized in Table 1.

Only a small proportion of the animals that caused the reported incidents were vaccinated (32% within the last 12 months since the incident, and 8% more than 12 months prior to the incident), which was in line with the estimated vaccination coverage achieved during the dog vaccination campaigns (mean=41%, SD=16% for each barangay across the study period). Both the dog vaccination coverage and patient bite incidence in each barangay were negatively correlated with the distance to ABTCs (Figures 3, 4A, B and Table 1). As such, barangays located further from the clinics were less likely to have had dog vaccination campaigns during the study period. This pattern also mirrors the distribution of the human population, with densely populated barangays aggregated in and around urban areas where the ABTCs were situated (Figure 4C). This result suggests that the dog vaccination campaigns are predominantly targeted at barangays with high population densities although the official strategy recommends prioritizing barangays with high numbers of dog rabies cases. Whether the higher bite incidence occurring in barangays closer to the clinics implies that health seeking behaviour is driven by increased rabies awareness (i.e., greater rabies awareness effort in urban settings) or rather discouraged by the distance required to travel to reach the clinics (or a combination of both) remains unclear.

Interestingly, while the relationship between the distance to clinics and laboratory confirmed cases identified through the study follow a similar pattern as with patient bite cases, the distribution of these dog rabies cases shows a wider spatial range (Figure 3B), suggesting wider spread and weaker links between urban centers and rabies transmission in dogs than observed for bite patient incidence.

### Impact of IBCM on Detection of Rabies in the Dog Population

In addition to the 22 dog rabies cases that were confirmed following investigations by the BITERS team, a further 27 dog rabies cases were confirmed at RADDL following submission by dog owners, independently of the BITERS team field efforts. Submissions handled by dog owners may have been encouraged by the increased awareness raised through the presence of the field team in the area. All of the laboratory confirmed cases were found nearby the BITERS investigation sites (Figures 3B, C). Overall, the case detection increased fourfold over the 13 months of this study, from 12 cases (0.007% of the dog population) confirmed in the previous 13 month period of routine surveillance, to 49 cases during the period with Integrated Bite Case Management (0.02%).

**Figure 5:**
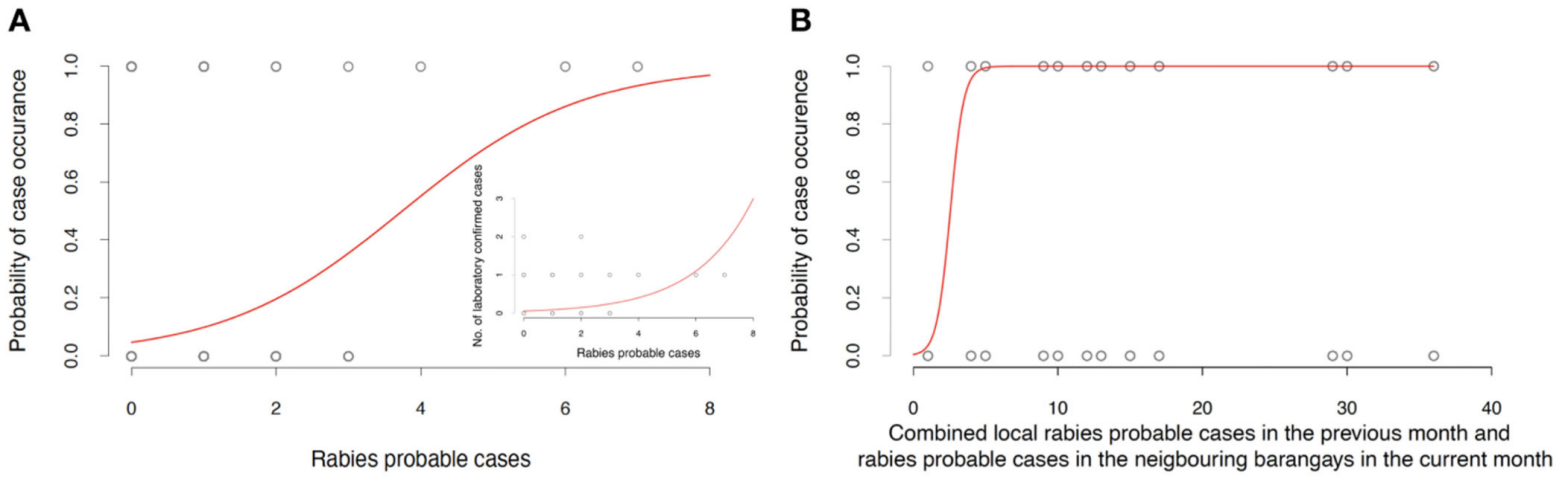
Validation and spatiotemporal analysis of the BITERS surveillance data. **(A)** Relationship between monthly “rabies probable” cases in each barangay and the probability of confirming rabies in a given month and barangay. The inset depicts the positive relationship between monthly “rabies probable” cases in each barangay and laboratory confirmed rabies cases in a given month and barangay. **(B)** Probability of rabies confirmation at barangay level increases with the number of “rabies probable” cases in the local barangay in the past month and “rabies probable” cases occurring currently in neighbouring barangays. Statistics of all reported models summarised in Table 1.

We found that the number of monthly “probable” rabies cases at the barangay level identified by IBCM strongly correlated with both the number of confirmed cases and the probability of case confirmation (Figure 5A and Table 1), providing convincing evidence that use of IBCM can increase rabies detection compared to routine surveillance in the Philippines. The results of binomial regressions to examine predictors of rabies cases suggest that rabies transmission happens mostly locally (as cases tend to be clustered in space and time) with cases in the focal (i.e., home) barangay from the previous month and current cases from neighbouring barangays most significantly predicting future rabies occurrence (Figure 5B and Table 1) in comparison to cases elsewhere in the province or the municipality.

## Discussion

Despite ongoing rabies prevention programs, the Philippines currently has both high per capita bite patient incidence and human rabies deaths within Southeast Asia (13) and worldwide (1). Here, we found that the patient bite incidence in Albay was high, with PEP administration reflecting vaccine availability and proximity of bite patients to clinics rather than the distribution of dog rabies cases. For example, all patients classified as WHO Category I exposures were administered at least one vaccine dose (with most completing a full course) despite such exposures not requiring vaccination according to WHO guidance (16). Similarly, costly RIG was often provided with substantial delays even though its purpose is to provide protection before the development of vaccine-induced immunity, and it should not be administered beyond day 7 after the initial vaccine dose (21). While consistent with previous reports of generous use of PEP and RIG in the Philippines (6, 22), this is an unnecessary expenditure, especially as evidence suggests rabies vaccines are highly effective even in the absence of RIG (21). Considering that >200,000 USD were spent on RIG over the 13 months of the study, reserving RIG only for Category III patients with multiple exposures, could in theory have saved sufficient funds to procure vaccine for the majority of the dog population in Albay province. This could potentially have greater impacts on public health through the interruption of dog rabies transmission.

ABTCs that provide rabies PEP have been established in every province across the Philippines, but the burden of bite patients falls disproportionately on clinics according to their accessibility and the socioeconomic situation of the region. Often, with free provision of PEP and seemingly unlimited stocks, systematic risk-assessment of patients is lacking both by healthcare providers and patients themselves, leading to substantial precautionary use of PEP (22, 23). In the Philippines, PEP use has been continually rising. This caused PEP shortages across the country in the second quarter of 2018 and was exacerbated by a wider global vaccine shortage (6, 23). In response to a PEP shortage, many patients were redirected to acquire post-exposure vaccines from private clinics at their own cost, precipitating additional inequity in vaccine access (22). Our results are in line with similar reports from other provinces across the Philippines, highlighting the urgency for discerning high-risk patients for PEP administration and to prevent unnecessary casualties from depleted vaccine stocks (6, 23). Whilst it is an ethical imperative to improve access to PEP for those at risk, indiscriminate PEP administration results in financial strains and vaccine shortages.

Moreover, the risk of rabies exposure persists as long as rabies continues to circulate in domestic dog populations and is highest for marginalized and hard-to-reach communities that tend to have lowest health-seeking behaviour (8, 24). Dog rabies cases are, however, severely underreported as a result of weak surveillance (8), which in turn leads to low prioritization of control programs, especially in settings where rabies competes with other diseases considered to be of greater economic importance. The surveillance protocol piloted in this study facilitated a fourfold increase in the detection of laboratory confirmed dog rabies cases. Our results suggest that such a targeted surveillance approach offers an effective way to locate foci of infection both to obtain better estimates of rabies incidence in dogs and to prevent further spread, through dog quarantine and increased rabies awareness.

The spatial pattern of the detected cases is likely related to the nature of rabies transmission. Rabid dogs typically bite other dogs within 1km of their homestead (18). Our analysis suggests transmission occurs in clusters; a barangay is predicted to experience a case with the highest probability if a “probable” case has been reported in a neighbouring barangay in the same month or locally a month prior. These results make sense given the length of rabies serial interval, but also have helpful management implications. Barangays that are experiencing rabies cases, as well as their neighbours, should be on guard for future transmission, adhering to enhanced surveillance, practicing risk-averse behaviours, quarantining all “rabies suspect” dogs and ensuring communities are aware of the risks. Where vaccination coverage is low and vaccines are available, reactive vaccination may provide some benefits, but will not be sufficient without annual comprehensive mass vaccination campaigns (25). Through the detection of circulating rabies cases, IBCM can also point to areas where routine dog vaccination needs strengthening.

Whilst it is impossible to discern whether the increased case detection resulted purely from improved surveillance or other causes, there is no evidence to suggest that the number of cases during the study period increased due to reduced vaccination coverage in this period. The heterogeneity of coverage may play a role in providing opportunistic pockets of susceptibility, shaping the distribution and size of outbreaks. However, given that the vaccination coverage in Albay province was consistently below the recommended 70% it is more likely that the increase in detected cases was a consequence of the improved surveillance. Additionally, incursions from nearby populations may have contributed to the burden of infection as can be expected in highly connected landscapes such as the Philippines. The number of viral lineages circulating within a region and their origin can be assessed by viral sequence data. Hence, incorporating genomic surveillance into IBCM would generate additional insights regarding the circulation of the virus and could inform the ongoing control program (26).

We conclude that the risk of rabies exposure is disproportionate depending on the geographic location and the socioeconomic background (27, 28), and that the physical access and cost of attending a clinic differs between communities. Integrated One Health approaches to rabies surveillance have potential to substantially increase case detection, and inform more judicious and cost-effective PEP provisioning, while proactively identifying areas and individuals most at risk who would otherwise not receive attention or seek care (29). Similar IBCM/enhanced surveillance studies have been piloted in different settings such as Haiti, Chad, Madagascar, and Tanzania (24, 30–33), generating highly valuable, context-specific information critical to building equitable healthcare systems in resource-limited settings. However, further implementation research is required to operationalize procedures for more judicious PEP administration as healthcare decisions are sensitive and challenging to put into routine practice.

The need to reduce the burden of rabies, and to implement interventions to minimize rabies risk is pressing, with a global strategy in place aiming to achieve zero human deaths due to dog-mediated rabies by 2030 (3). With short-lived or sparse resource allocation for rabies surveillance and control, low-cost approaches tailored to local settings are needed. Here we showed that IBCM offers a cost-effective tool for increased and earlier detection of rabid dogs (by using patient bite-histories to inform field investigations), insights into potential improvements of PEP provisioning that could reduce unnecessary expenditures (by using patient bite-histories and field investigations to inform healthcare decisions) and potential guidelines for strengthened dog vaccination (by using insights generated by analysing the above data to inform canine rabies management). We further advocate for intersectoral collaboration and community participation, including collaborative investments between public health and veterinary services, as the foundation of sustainable and equitable One Health practice.

## Figures and Tables

**Table 1:** Summary statistics for BITERS regression analyses. All variables are calculated at the barangay level.

Response variable	Explanatory variable	Model regression	p-value	coefficient	residual deviance	DF	AUC	Graphic
Patient bite incidence	Distance to ABTC	Gamma	< 0.005	0.007	1213.3	718	–	Figure 4A
Dog vaccination coverage	Distance to ABTC	Linear	< 0.005	-0.008	14.7	718	–	Figure 4B
Dog vaccination coverage	Distance to ABTC truncated to 30 km	Linear	< 0.005	-0.006	12.7	674	–	–
Dog vaccination coverage	Human population density	Linear	< 0.005	1.075 e-05	18.6	718	–	Figure 4C
Human population density	Distance to ABTC	Gamma	< 0.005	8.912 e-05	549.82	717	–	Figure 4C inset
Probability of confirming rabies in the given month and barangay	Monthly rabies probable cases	Logistic	<0.005	0.807	317.84	717	0.63	Figure 5A
Laboratory confirmed rabies cases in the given month and barangay	Monthly rabies probable cases	Poisson	<0.005	0.501	252.77	717	–	Figure 5A inset
Probability of confirming rabies in the given month and barangay	Rabies probable cases in the local barangay in the past month and current rabies probable cases in neighbouring barangays	Logistic	<0.005 (for both variables)	1.4757, 0.657	591.7	9344	0.6	Figure 5B

## Data Availability

The anonymised data supporting the conclusions of this article will be made available by the authors, without undue reservation.

## Footnotes

**Supplementary Material**
